# Endodontic Management of Maxillary Second Molar Tooth with a Single Root and Single Canal

**DOI:** 10.1155/2020/2829304

**Published:** 2020-02-01

**Authors:** Neelam Mittal, Vijay Parashar, Prasad Suresh Patel

**Affiliations:** Faculty of Dental Sciences, Institute of Medical Sciences, Banaras Hindu University, Varanasi, Up, India

## Abstract

Comprehensive understanding of variations in the root canal morphology of a maxillary molar is useful for performing successful endodontic treatment in such cases. This case report describes endodontic management of a case with such aberrant root canal morphology of a maxillary second molar having a single root and single canal.

## 1. Introduction

The variations in root canal anatomy of multirooted teeth are a constant challenge for diagnosis and successful endodontic management [1]. Thus, it is necessary for a clinician to have thorough knowledge of variation in root canal anatomy of multirooted teeth. A maxillary second molar usually has three roots and three canals. The maxillary molar has been reported with various root canal anatomy having four roots [2], two roots [3, 4], and even a single root with a single canal [5]. The prevalence of a single canal in the maxillary molar is reported to be 0.5-0.6% [2, 5].

Fava et al. [6] have reported a study on morphologic variation in maxillary and mandibular molars. They have reported variations in the root canal system having a single root and single canal in maxillary and mandibular molars; however, it was reported to be most frequent with the mandibular molar. The majority of variations seen in the maxillary molar are seen with the maxillary first molar. Variations in root canal anatomy of the maxillary second molar are quite rare.

Kim et al. [7] in their study using CBCT found the incidence of a single-rooted maxillary second molar to be 10.7% in the Korean population, and Zhang et al. [8] found the incidence to be 10% in the Chinese population. However, study on the Indian population by Neelakantan et al. [10] reported 0.9% of maxillary second molars with a single root but none with a single root canal.

Use of multiple angled radiographs and advanced radiographic diagnostic methods such as cone beam computed tomography (CBCT) or use of magnifying loupes or operating microscope can lead to appropriate diagnosis and management of such complex root canal anatomy [8].

The aim of the present paper was to report an aberrant root canal anatomy with the maxillary second molar.

## 2. Case Report

**Figure 1:**
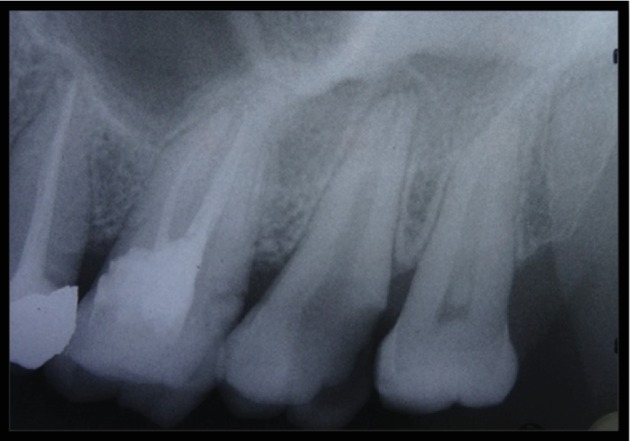
Preoperative IOPA.

A 32-year-old female reported to the Department of Operative Dentistry and Endodontics, Banaras Hindu University, Varanasi, India, with a chief complaint of pain in the upper left back tooth, which she reported was increasing while biting and taking cold or hot beverages. The patient's medical history was noncontributory. On clinical examination, there was deep proximal caries with the maxillary left second molar; thus, intraoral periapical radiograph (IOPA) of tooth 27 was advised. The tooth was nonmobile, and periodontal probing was also within the physiological limit. Radiographic examination revealed the carious exposure of tooth 27, and thus, root canal treatment of the teeth was advised. Radiographic examination also revealed some variation in root canal anatomy with tooth 27, and having a single root and single canal (Figure 1) was suspected.

**Figure 2:**
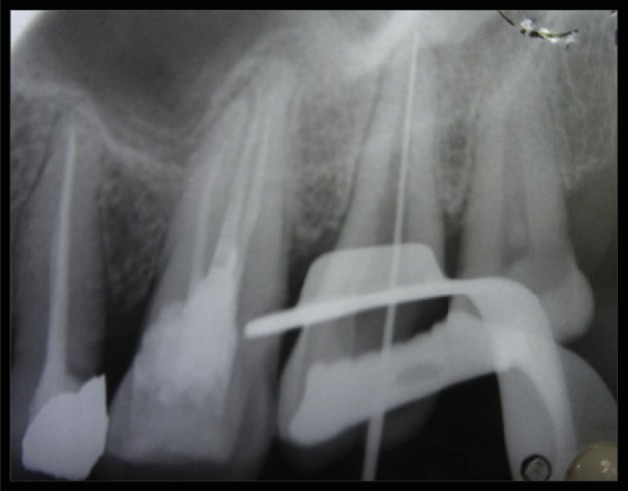
Working length IOPA.

The tooth was anesthetized with 1.8 mL (30 mg) 2% lignocaine containing 1 : 200,000 epinephrines (Xylocaine; AstraZeneca Pharma Ind Ltd., Bangalore, India.) followed by rubber dam isolation. Access opening with the aid of magnifying loupes (zumax 3.5X) of tooth 27 revealed a large single orifice and one single wide root buccolingually extending toward the root apex. Working length (WL) was determined with an apex locator (CanalPro Apex Locator, Coltene) and was confirmed with k-file (Figure 2). WL IOPA also confirmed the single-rooted maxillary second molar with a single canal. Root canal shaping was done with k-files using the conventional method of canal preparation and was irrigated with sodium hypochlorite (Sigma Aldrich, Germany). EDTA was used to remove the inorganic component of the root canal system, and a smear layer formed with filing the dentin. Saline was used as intermittent irrigation. Due to the wide nature of the canal, more attention was paid on chemical disinfection.

After canal preparation was done, IOPA was taken to confirm the fit of the master gutta percha cone. A lateral condensation technique of obturation with Apexit Plus as a sealer was used.

**Figure 3:**
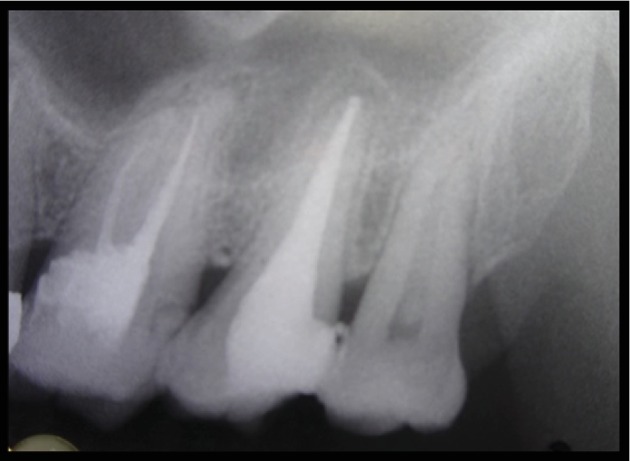
Post obturation IOPA.

**Figure 4:**
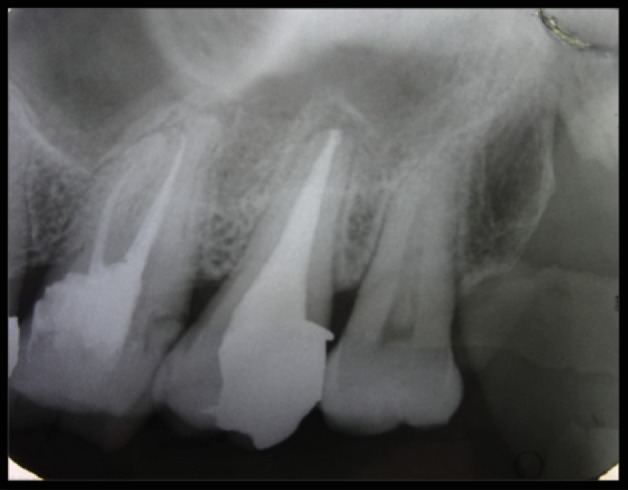
Final restoration IOPA.

Post obturation, IOPA was taken to examine filling of the root canal system (Figure 3). Follow-up IOPA two weeks after (Figure 4) was taken, and final restoration with amalgam was done, and the patient was referred to the Department of Prosthodontics for further treatment.

## 3. Discussion

Such variation in the maxillary molar having a single root and single canal can be easily detected in routine radiograph. However, multiple preoperative radiographs can help us differentiate if two canals are present superimposing each other buccolingually. Use of an advanced diagnostic method such as CBCT can help rule out complex root canal anatomies where radiograph will be inconclusive.

Variation in root canal anatomy of the maxillary molar having a single root and single canal is reported less frequently. Literature review reported the incidence of the maxillary second molar having a single root and single canal to be 0.5-3.1% [2, 5, 9].

Study on the Indian population reported the incidence of 0.9% having a single root in the maxillary second molar, but none with a single root canal were reported [10].

The search for a missing canal can lead to common iatrogenic mishaps such as perforation or excessive tooth removal. Such error can be avoided if a clinician has general idea of variations present in the root canal system. This can be achieved with thorough preoperative evaluation of the root canal system with multiple angled root canals and use of CBCT when conflicting opinion presents about the complex root canal system. Use of magnifying loupes or a dental operative microscope can be very effective in such cases to perform ideal root canal treatment.

Various studies are reported in vivo and in vitro about the variations in root canal anatomy of the maxillary and mandibular molars.

Libfeld and Rotstein [2] in their study reported an incidence of 0.5% out of 200 radiographs of patients treated with endodontic treatment having maxillary second molars with a single root and a single canal in their in vivo study. Rwenyonyi et al. [11] also found a single root in a maxillary molar; however, the roots were found to be fused.

Wang *et al*. [12] in their study reported that the incidence of a maxillary second molar with a single root and a single canal is very rare. Ng et al. [13] and Alavi et al. [14] failed to find a case in their 77 maxillary second molars with a presence of a single root and a single canal.

Thus, these studies suggest that the incidence of a maxillary molar having a single root is not high. And so, it becomes important to recognize such cases while performing the endodontic treatment to avoid iatrogenic procedural errors.

## 4. Conclusion

Knowledge of variations in root canal anatomy guides in designing the therapy to be implemented in a particular case and can also affect the possibility of success with that therapy. Management of a maxillary second molar with a single root starts from proper diagnosis and modification in the treatment strategy accordingly. Therefore, practitioners need to be well equipped with knowledge of abnormalities in molar teeth and the prevalence of those abnormalities.
